# Primary pancreatic leiomyosarcoma: a case report

**DOI:** 10.1186/1757-1626-1-280

**Published:** 2008-10-28

**Authors:** Shams Ul Islam Muhammad, Faisal Azam, Stokes Zuzana

**Affiliations:** 1 Department of Medical oncology, Churchill Hospital, Oxford, OX3 7LJ, UK; 2 Department of Clinical Oncology, United Lincolnshire Hospitals NHS Trust, Lincoln, LN2 5QY, UK; 3 Department of Medical oncology, Churchill Hospital, Oxford, OX3 7LJ, UK

## Abstract

**Background:**

Leiomyosarcoma are malignant tumours of the soft tissues. It is most commonly found in the stomach, small intestine and retroperitoneum. Primary pancreatic leiomyosarcoma is extremely rare and only 22 cases have been reported in the literature since 1951. Its prognosis is poor and the palliative treatment is only aimed at symptomatic improvement.

**Case presentation:**

We report case of primary pancreatic leiomyosarcoma with liver metastases in a 73 year old man who presented with weight loss, epigastric pain, anorexia, abdominal fullness and obstructive jaundice. Histological examination and Immunohistochemical analysis of liver biopsy specimen confirmed the diagnosis of metastatic leiomyosarcoma. The patient died of disease 3 months after the initial diagnosis.

**Conclusion:**

Leiomyosarcoma of the pancreas is an extremely rare malignancy with a poor prognosis. Treatment is aimed at symptomatic improvement and pain management.

## Background

The incidence rate for pancreatic cancer is approximately nine new cases per 100,000 people, with the peak incidence in the seventh and eighth decades of life and an average age of 60 to 65 years at diagnosis [1]. The commonest type is an adenocarcinoma.

Primary pancreatic leiomyosarcoma is a rare malignant neoplasm. It accounts for 0.1% of malignant pancreatic cancers [1]. A review of the literature reveals 22 such cases published so far [2]. The majority of the reported cases were diagnosed on autopsy.

Survival in patients with untreated PC is poor. For all stages combined, the 1-year survival rate is 19% and the 5-year survival rate is 4% [1]. The majority (80%) of PCs are metastatic at the time of diagnosis. Surgical resection (when margin negative, node negative) offers the best possibility for cure, with 5-year survival approaching 40% when performed at specialized major medical institutions [3].

We report a case of Primary leiomyosarcoma of Pancreas occurring in a 73 years old male.

## Case presentation

A 73 years old male presented with 1 month history of weight loss of 16 kilograms, epigastric pain, anorexia, abdominal fullness and jaundice. He was recently diagnosed with diabetes mellitus. Liver was palpable on examination of his abdomen. He had a raised bilirubin and liver enzymes. Abdominal ultrasound & CT scan demonstrated an enlarged liver containing multiple irregular ill defined lesions including the caudate lobe suggestive of metastases. There was a large enhancing soft tissue mass with a central necrotic area approximately measuring 9.4 × 10 cms in the region of the body of pancreas. This mass was compressing splenic artery and vein. The patient underwent Ultrasound guided liver biopsy. Histological & Immunohistochemical analysis confirmed leiomyosarcoma of the pancreatic origin. This case was discussed in relevant multidisciplinary team meeting. As his disease was already in advanced metastatic phase so he was offered palliative chemotherapy. His condition deteriorated before the chemotherapy & he died after 3 months of the initial diagnosis. No autopsy was conducted.

**Figure 1:**
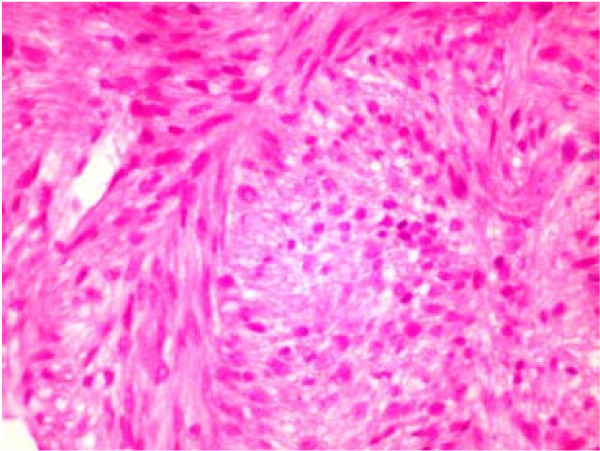
Photomicrograph of the liver biopsy stained with Haematoxylin and Eosin (H&E) Original magnification × 200.

**Figure 2:**
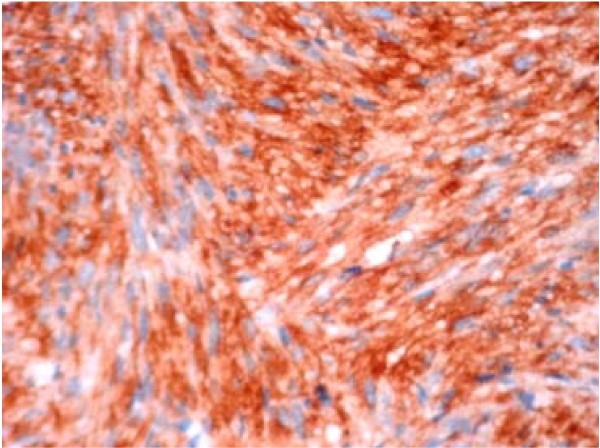
Photomicrograph of the liver biopsy stained with smooth muscle actin (SMA) Original magnification × 200.

Histological examination was suggestive of Spindle cell neoplasm (Figures 1 &2). The spindle cells showed mild to moderate nuclear pleomorphism & anisocytosis. There was generally less than 1 mitotic figure per high power field & no abnormal mitotic figures were seen. The spindle cells were positive for desmin & smooth muscle actin. These were negative for cytokeratin (AE1/AE3), HMB45, S100 protein, melanin A, NSE (neuron specific enolase), CD34 and CD 117. The immune profile was suggestive of leiomyosarcoma.

In conclusion, the histological & Immunohistochemical features of the tumour were consistent with low to intermediate grade leiomyosarcoma.

## Discussion

Primary pancreatic leiomyosarcoma is a rare malignant neoplasm reported in world literature by only 22 cases up to this time.

We report a case of primary pancreatic leiomyosarcoma. This tumour was involving the body of the pancreas with direct invasion of the splenic vessels along with multiple hepatic secondaries. Complete clinical and radiological evaluation excluded secondary pancreatic involvement by a sarcoma of the retroperitoneal and gastrointestinal origin. Immunohistochemical analysis of the samples confirmed this tumour as a smooth muscle sarcoma.

Leiomyosarcomas involving pancreas consist of primary pancreatic leiomyosarcoma or pancreatic extensions of retroperitoneal and gastro-duodenal sarcomas [2]. Pancreatic mesenchymal tumours consist of malignant peripheral nerve sheath tumours, malignant fibrous histiocytomas, liposarcomas, rhabdomyosarcomas, hemipericytomas & leiomyosarcomas [3]. A detailed clinical evaluation is essential to rule out a primary extra pancreatic leiomyosarcomas. Female genital tract, gastrointestinal tract, soft tissues of the extremities and retroperitoneum are the most commonly affected areas [2].

Table 1 summarizes the clinicopathological data of the 22 reported cases of pancreatic leiomyosarcoma. Abdominal pain, weight loss, epigastric tenderness & abdominal mass are the most commonly presenting symptoms [4]. Pancreatic leiomyosarcoma is more common in fifth decade of life or older (mean age 44.7 years; range 14 to 80 years). Males are affected almost twice as compared to females (7:4). The size of the tumours is quite variable ranging from 3 cm to 25 cm (Median,10.5 cm). It is observed that larger tumour can develop cystic degeneration & can be misdiagnosed as pseudocysts of pancreas [4].

**Table 1:** Clinicopathological data of reported cases.

**Case Number**	**Year**	**Author**	**Age/Sex**	**Clinical features**	**Morphological Findings**	**Clinical management & outcome**
1	1951	Ross	80/M	Weight loss, mass, LUQ	Whole pancreas; wide spread metastases without lymph node involvement	Autopsy case
2	1956	Berman & Levene	47/M	Jaundice	Head of pancreas;5.5 cm; no metastases	Pancreatodudenectomy ; well 1 year later
3	1957	Feinberg et al	14/M	Epigastric pain; nausea	Head;11 cm; no metastases	Pancreatodudenectomy
4	1965	Becker et al	No data available	No data available	Cystic change	No data available
5	1970	Oyamada et al	47/M	General fatigue, mass, LUQ	15 cm	Nonresectable
6–10	1973	Baylor & Berg (5 cases)	51 (Median), M:3, F:2	No data available	Localized:1;Locally advanced:1;Disseminated:3	No data available
11	1976	Carda et al	56/F	Nausea & vomiting	Whole pancreas	Gastrostomy; died in 9 months
12	1981	Ishikawa et al	44/M	Epigastric pain, mass	Head;8 cm,; localized at operation	Pancreatodudenectomy; liver metastases 4 years later
13	1982	Tulha et al	28/F	Weight loss, mass	Head;20 cm	Pancreatodudenectomy ; Lung & liver metastases 15 months later
14	1990	Murata et al	55/F	No data available	Head & tail; no metastases	Caudal pancreatectomy
15	1991	Lakhoo & Mannell	68/M	Abdominal pain, weight loss, mass	Body;17 cm; no metastases	Distal pancreatectomy, gastric resection & transverse colectomy; well 2 years later
16	1993	De Alava et al	71/M	Abdominal pain, weight loss	Body;3.6 cm; no metastases	Pancreatectomy
17	1994	Peskova & Fried	68/F	Malena	Head;15 cm; localized	Pancreatodudenectomy;well 3 years later
18	1994	Sato et al	53/F	Abdominal pain, mass	Body;25 cm; no metastases	Distal pancreatectomy
19	1994	Ishii et al	66/M	Incidental finding	Tail;14 cm; wide spread metastases without lymph node involvement	Nonresectable
20	1995	Aranha et al	46/F	Epigastric pain, nausea, vomiting	Body;3 cm; localized	Distal pancreatectomy; dead in 9 months
21	1997	Owen et al	40/M	Abdominal pain	Head;6 cm; no metastases	Pancreatodudenectomy; well 10 year later
22	2000	Nesi et al	76/M	Fever	Tail;8 cm; localized	Distal pancreatectomy; dead in 12 months
23	2007	Present case	73/M	Weight loss, Epigastric pain, abdominal mass	Body;10 cm, hepatic metastases	Nonresectable; dead in 3 months

In the documented literature, about 43.5% (10 patients) presented with locally advanced or metastatic disease. First ever case of pancreatic leiomyosarcoma was diagnosed on autopsy [5]. Three patients died at 3,9 and 33 months respectively after the diagnosis [6,7]. We do not know precisely about the survival outcome of remaining 6 patients.

At the time of diagnosis of leiomyosarcoma, only 13 cases seem to be localised to pancreas & underwent surgical resection. Followup details are available for only 8 patients. Four patients died of their tumours with in four years time after the initial diagnosis [6,8,9]. Remaining 4 patients were alive & free of symptoms at 1,3,4,6 years after the initial surgical management [10-12].

Large tumour size is not a precise predictor of adverse outcome. In leiomyomatous tumours mitotic figures are proposed to present the important predicting parameter [13]. Mitotic counts of more than 10 mitoses/10 HPFs is associated with a worse outcome [2].

## Conclusion

The number of cases of pancreatic leiomyosarcoma reported in world literature is limited but what we have learnt that it is a highly malignant tumour which can be metastatic even on first presentation. Extensive surgical resection of tumour is still the best treatment option for localised disease. Metastatic pancreatic leiomyosarcomas have shown only a partial response to chemotherapy.

Treatment is aimed at symptomatic improvement and pain management.

## Consent

Written informed consent could not be obtained since the patient is deceased and the next of kin were untraceable. We believe this case report contains a worthwhile clinical lesson which could not be as effectively made in any other way. We expect that the next of kin would not object to the publication since the patient remains anonymous.

## Competing interests

The authors declare that they have no competing interests.

## Authors' contributions

SIM & FA carried out the literature search and drafted the manuscript. SZ approved the version to be published. All authors read and approved the final manuscript
